# Comfort experience in palliative care: a phenomenological study

**DOI:** 10.1186/s12904-016-0145-0

**Published:** 2016-08-02

**Authors:** Adriana Coelho, Vitor Parola, Miguel Escobar-Bravo, João Apóstolo

**Affiliations:** 1 Nursing School of Coimbra, Coimbra, Portugal; 2 University of Lleida, Faculty of Nursing and Physiotherapy, Lleida, Spain; 3 Health Sciences Research Unit: Nursing, Nursing School of Coimbra, The Portugal Centre for Evidence-Based Practice: an Affiliate Centre of the Joanna Briggs Institute, Coimbra, Portugal

**Keywords:** Palliative care, End of life care, Inpatients, Qualitative research, Phenomenology, Comfort

## Abstract

**Background:**

Palliative care aims to provide maximum comfort to the patient. However it is unknown what factors facilitate or hinder the experience of comfort, from the perspective of inpatients of palliative care units. This lack of knowledge hinders the development of comfort interventions adjusted to these patients. The aim of this research is to describe the comfort and discomfort experienced by inpatients at palliative care units.

**Methods:**

A phenomenological descriptive study was undertaken. Ten inpatients were recruited from a Spanish palliative care unit and seven from a Portuguese palliative care unit. Data were collected using individual interviews and analysed following the method of Giorgi.

**Results:**

Four themes reflect the essence of the lived experience: The Palliative Care as a response to the patient’s needs with advanced disease, attempt to naturalize advanced disease, confrontation with their own vulnerability, openness to the spiritual dimension.

**Conclusions:**

Informants revealed that they experience comfort through humanized care, differentiated environment, symptomatic control, hope and relationships. The discomfort emerges from the losses and powerlessness against their situation. Even if such findings may seem intuitive, documenting them is essential because it invites us to reflect on our convictions about what it means to be comfortable for these patients, and allows incorporating this information in the design of focused interventions to maximize the comfort experience.

**Electronic supplementary material:**

The online version of this article (doi:10.1186/s12904-016-0145-0) contains supplementary material, which is available to authorized users.

## Background

The development of science and technology is expressed in an increase in life expectancy [1]. Therefore the population’s aging in a society in which death may be delayed ever more, allows us to predict a gradual increase in the prevalence of degenerative and disabling diseases. Also in recent years the process of dying was displaced from the home environment for the hospital context [2].

In this sense, there has been an increment of palliative care units (PCUs) with the aim of provide the greatest comfort and dignity possible to patients and their families facing the problem associated with life-threatening illness [1, 3].

However, patients with advanced disease still experience discomfort [4]. This could be explained by the fact that these patients often have comfort needs that extend beyond physical symptoms management [5]. Nevertheless the literature continues to give more attention to the physical comfort [6–8], and little attention is given to other aspects of comfort commonly observed among these patients [9].

Indeed, Kolcaba [10], defines comfort as the immediate experience of being strengthened by having the needs for three types of comfort (relief, ease, or renewal) met in four contexts of human experience (physical, psychospiritual, environmental, and social).

In this sense, knowing the experiences of comfort and discomfort of patients are a relevant aspect for the practice of care, guiding the care provided for the patients’ needs and maximizing the effect of comfort interventions.

Some researchers have sought to understand the comfort experience in the view of patients in other contexts [11–14]. These studies are important efforts to the comprehension of the phenomenon. However, in the context of Palliative Care (PC), such investigations are scarce [8]. There are only studies that describe this experience, in some of the contexts of comfort, but from the professional [15–18], or families perspective [19, 20]. However, it is important to note that both family and health professionals tend to describe the physical [21–24], and emotional symptoms [21, 25–27], differently to the patient. Therefore it is not clarified what is the patient comfort experience, and known that the patient’s comfort is an important objective of PC, their comfort experience should be taken into account.

Furthermore, the analysis of the literature evidence that the comfort interventions in PC are intuitive or based on medical principles [28].

Therefore being comfort/discomfort subjective states that can only be understood in the light of the patient’s experiences, starting from a concrete reality [29], and with the conviction that intervention processes must take into account the complexity and subjectivity of the patient experience, it was conducted this study in order to describe the comfort and discomfort experienced by inpatients at PCUs.

## Methods

### Study design

The present study is a secondary aim of a larger project about confort interventions.

This study was conducted using a qualitative phenomenological descriptive design. A descriptive phenomenology was chosen, in order to study the complex phenomenon of human experience, giving emphasis to how the life-world is described by the participants voices [30].

This study conforms to Consolidated Criteria for Reporting Qualitative Research (COREQ) guidelines (see Additional file 1).

### Participants and setting

Study participants were recruited from a Portuguese and Spanish PCUs, between March and May 2015.

The heads nurses, invited face-to-face those who were eligible to participate (Table 1).

**Table 1:** Inclusion and exclusion criteria

Inclusion criteria	Exclusion criteria
- Adult patients with incurable and advanced disease;	- Patients with cognitive alterations;
- Able to consent;	- Dying patients.
- Able to speak Spanish or Portuguese;	
- In health conditions that allow them to tolerate an interview of at least 20 min;	
- Stay period in the PCU equal or superior to 3 days.	

A purposive sampling strategy was performed to ensure a sample that included a wide spectrum of participant gender, ages, hospitalization time, and diagnoses [31].

A total of 17 inpatients participated (Table 2).

**Table 2:** Participants

		Spanish PCU	Portuguese PCU	Total
Gender	Male	5	3	8
	Female	5	4	9
Age		Range: 58–90 years	Range: 56–78 years	Mean: 70.5
		Mean: 74 years	Mean: 67 years	
Hospitalization time		Range: 4–44 days	Range: 9–76 days	Mean: 22.5
		Mean: 14 days	Mean: 31 days	
Diagnoses	Oncologic	7	7	14
	Non-oncologic	3	0	3

### Data collection

Data were collected through non-structured interviews. Interviews were chosen taking into account the vulnerability of participants [32]. Furthermore this technique facilitates a personal narrative by the participant [33].

Non-structured interviews were conducted, supported by the original question: How did you live the experience of being hospitalized in this unit?, with the intention of the significant experience of comfort and discomfort to emerge freely.

Follow-up questions in order to deepen understanding of the experience of the informants were also carried, such as: How would you describe this in more detail? What does that mean to you?

A pilot test with two patients was conducted in order to adjust the interview question. These interviews were not included.

Interviews were individual, mean duration was 32 min, and were held in a location of the participants’ choice (their room or an intimate space in the PCU). They were digitally audio-recorded and transcribed verbatim.

It was assumed that saturation had been reached after the 10 Spanish PCU and 7 Portuguese PCU interview. Non-participants refused to participate in the study or dropped out during the interview.

Data collection was carried exclusively by one of the investigators (AC) in order to avoid significant differences in conduction the interview. Transcripts were reviewed by the interviewer (AC) to verify their accuracy.

The findings were not returned to participants for confirmation because of participants’ declining health. The patient’s vulnerable conditions constituted a limitation to the rigor of the study, since it was not possible to confirm the findings with the interviewees. Thus, during the interviews were performed cross-checks to clarify and confirm the coherence of the mentioned for patients in their reports.

### Data analysis

Consistent with Giorgi method [30, 34, 35], analysis involved four steps. The first step was a reading of transcripts, several times, to get a sense of the whole experience. This was done without a critical reflection on the experience. Posteriorly, in the second step, was performed a subsequent readings of the transcripts with the purpose of identify the meaning units (seccions of the collected data that could reveals potentials aspects of the phenomenon under investigation). Each meaning unit is delimited by a change in the thematic content.

In third step the delineated meaning units identified in the previous step were transformed in appropriate language to the phenomenon under study and grouped into common themes and sub-themes that represent the essence of comfort experiences. In this step, the researchers performed imaginative variation by changing qualities of the object under analyzed so as to determine which data are essential. The imaginative variation permitted to determine the essence of the phenomenal structure of the experience.

The fourth analysis step consisted in synthesize all of the transformed meaning units into a consistent and descriptive statement regarding the subject’s experience of confort.

According to Giorgi [30, 34, 35], how or where the meaning units are delineated is not absolute, different researchers may delineate the meaning units in different places in the same data. To ensure rigour, each authors performed individual analyses. Every step of analysis were compared and discussed to strengthen the validity of analysis.

To manage the data was used the QSR NVivo version 10 software.

### Ethical considerations

Ethical approval was obtained by the Research Ethics Committees of the Fundació d’Osona per a la Recerca i l’Educació Sanitària (reference 2015873), Arcebispo João Crisóstomo Hospital (04 February 2015) and Health Sciences Research Unit: Nursing (reference 228–10/2014). Participating organisations’ ethical requirements were met.

The interviewer works in the Spanish Center; however during the data collection period did not work in the PCU. Participants were not acquainted to the researchers prior to the study commencements. They were made aware of the aim of the study, place of work and role of interviewer to inform their decision-making.

Participation was voluntary and they were informed of their right to withdraw from the study at any time. Complete confidentiality was guaranteed and a written consent was obtained by the main researcher before each interview.

## Results

**Fig. 1:**
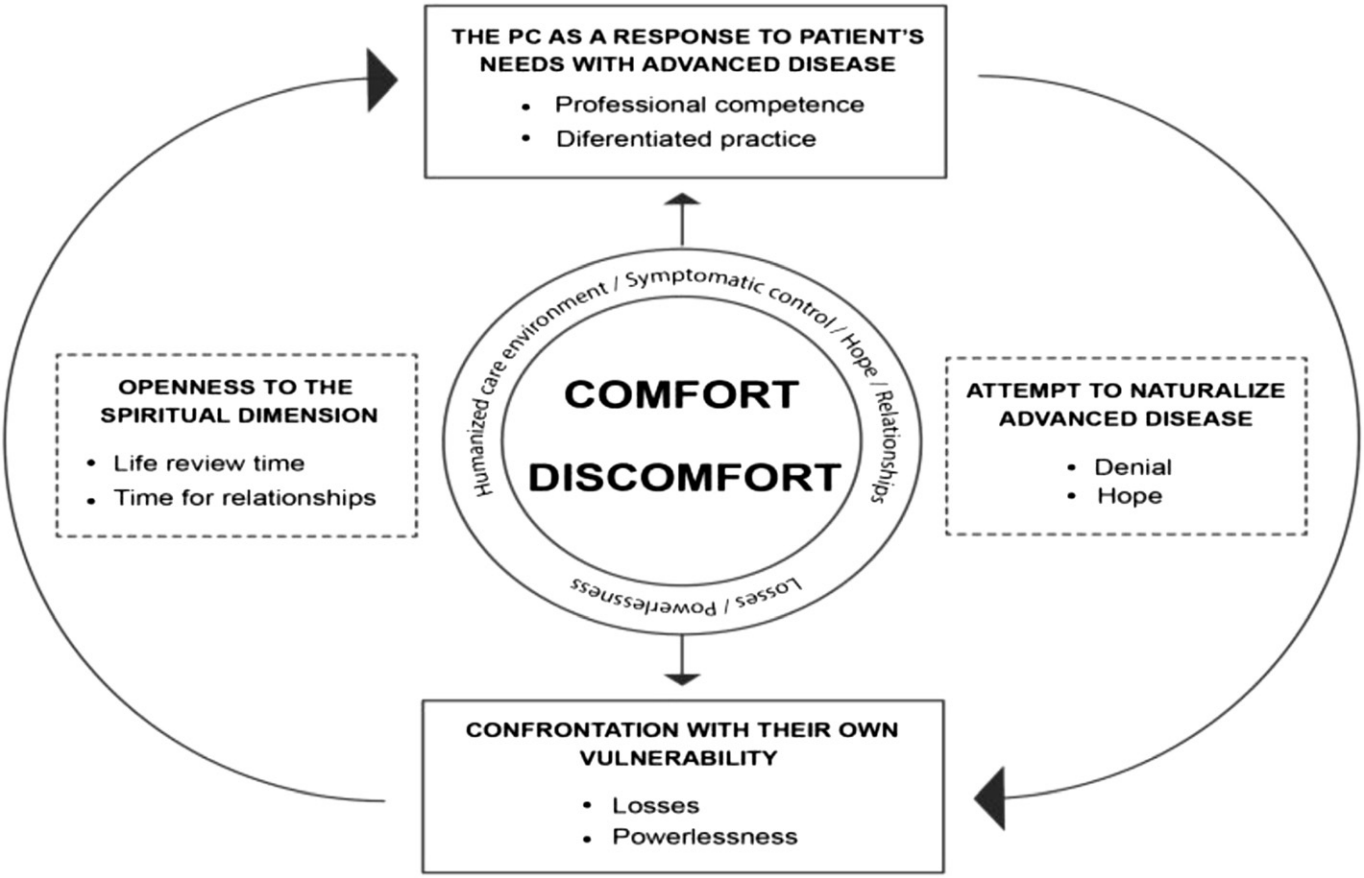
Conceptual representation of the sub-themes and themes based on the findings

The analysis of the findings allowed the access to a comprehensive scheme (Fig. 1) organized in an interactive structure.

### The PC as a response to patient’s needs with advanced disease

Informants acknowledge that, to find responses to their health care needs, they need to be hospitalized in a PCU:

*P16 “(…) the pain and the shortness of breath, that is the thing I was not able to control, and that was creating some fearful respect. At the hospital I am well, arriving home, I stay there for a little longer and that’s it…loss of control.”*

The PCU is perceived as a relief space of physical discomfort, but also a space of comfort by the human competence, by the surrounding environment and by rapidly attention to the patient’s needs:

*P12 “If you need a nurse, a nurse will come immediately. If you need anything, just call them and they will come here straightaway. It is the comfort of knowing that if I need to call the nurses, they come here immediately.”*

By making reference to these comfort factors, the informants establish constant comparison with the experience lived in other services in which they were previously hospitalized:

*P10 “I had never been in a facility with such human quality as this one. Here the staffs are attentive to everything, I mean everything. And always smiling”.*

*P9 “It is a fact that here I have practically the same things as in the other hospital, my blood pressure is assessed, my medication is given to me. Nevertheless, here, besides it, there is a place where I can go down and go for a walk.”*

### Attempt to naturalize advanced disease

Symptomatic control obtained through the hospitalization in PCU leads to some informants naturalized the advanced disease, denying the proximity of death:

*P1 “(…) this is a temporary situation of one or two months which will end. This situation does not worry me, everyday I improve greatly and I am very positive.”*

In this sense, they described a sense of hope regarding recovery and return home.

*P15 “My hope is to recover the least independence which I know is possible, so I can go home to my little dog and to my kittens.(…) I believe that there is always a tomorrow, that there is hope.”*

The informant reports also reveal the conviction that it is possible to interfere with the course of the disease by the optimism:

*P14 “By saying: I am fine! The disease goes away quicker … “*

### Confrontation with their own vulnerability

Even if the PCU be recognized as an area that provide comfort, it is also seen as a discomfort space, where physical and social losses, the feeling of helplessness, confronts informants with their vulnerability and finitude.

*P3 “The problem that I now have is a breathing one… I have difficulty even speaking…my body has given all it has got”*

*P1 “Always the bedpan…I feel dependant. I am always waiting for someone to do everything for me.”*

*P17 “(…) watch the land, listen to the birdies and look at the things I have…the yards, the trees… until this got to me in a stronger way and everything ended. To be comfortable is not to have anything, not to have any problem, it is to be at my home.”*

Informants of Portuguese PCU even describe as uncomfortable experience the loss of freedom, which corresponds with the lack of an exterior space intended for patients (this space exists in Spanish PCU and was described as comfort proportioning)

*P17 “This means a jail. It’s a prison to be locked here, a prison.”*

In the Spanish context, the loss of freedom, for some informants it relates to the fact that they have to share a room with another patient.

*P3 “I have a neighbour who wants the blinds always closed…I like to read the newspaper and because of that I can’t read and that also makes me feel uncomfortable.”*

The experience of all these losses generates impotence and devaluation feelings on the informants:

*P14 “I feel inferior… I wanted to go alone (to the toilet) but I can’t. Asking for help means wanting and not being able to.”*

Due to those experienced losses and powerlessness that they generate, some informants sense the imminence of the end of life:

*P17 “That was why I was sent here… perhaps it was so that I could end my days in here… “*

### Openness to spiritual dimension

The confrontation with their own vulnerability raises in some patients the opening to the relationship intrapersonal, to the need to do a review of his personal history.

*P14 “I believe I have not affected anybody…here we think about everything, we weigh everything, and here some things are left to be concluded and others left halfway.”*

This time dedicated to the life review allows the revaluation of what is really important.

*P10 “I lost too much time scolding my family…and today I can only think of that everything we lived was wonderful… We don’t regard things until they are lost.”*

During this process the world of relations contains a special importance. Thus, from the speech of the participants emerges comfort that comes from affection with peers, with friends, with family and with the professionals themselves.

*P6 “I have a perfect partner. That means I can share with him my problems, that I can trust him as if he was my family.”*

*P15 “It is to feel loved, to feel nurtured, to feel spoiled.” (have friends visit)*

*P17 “There is a moment which is special, which is my wife’s visit who comes here everyday.”*

*P9 “This team that cares us so well, with such kindness… I think that for a patient it is as important a good medication or treatment as it is a humanised and nurtured care.”*

The speech of patients also points to the comfort that comes from the relationship with the transcendent – with Nature and with God.

*P9 “To be able to go outside for a walk is to inflate an internal joy which makes me feel alive.”*

*P17 “It is him (God) who comforts me, he is my saviour.”*

## Discussion

In previous research under the comfort in PC, comfort often appears associated with the physical dimension of the person [7, 36, 37]. However, this study showed that in addition to physical symptoms, there are other factors that promote significantly comfort and discomfort experiences of inpatients in PCU that should be taken into account.

Therefore, informants recognize as comfort sources, technical competence and human competence with which they feel care in PCU.

The findings suggest that although some comfort interventions seem simple and of little technological complexity (such as availability, fondness, support), they had the ability to significantly affect the state of comfort.

Informants also make a clearly positive assessment of care received in PCU compared to other units in which they have already been admitted, stating that the PCU is a different human and environmental structure. Kolcaba [38, 39], to define environmental comfort makes reference to the environment and to internal and external conditions such as noise, light, temperature or natural elements.

Previous researches report the environment of the PCU [40, 41]. However, this study brings new data since patients emphasize the PCU environment as evidence of the comfort experience through the external environment adjusted (Spanish context) and reduced noise (Portuguese context). The setting was also described as a comfort factor in the study of Hamilton [42], reported however to the presence of homelike elements to patients.

The satisfaction of needs that required hospitalization, leads to some patients try to naturalize advanced disease and make a denial of the proximity of death.

According to Kubler-Ross “denial is usually a temporary defence and will soon be replaced by partial acceptance” [43] (p.39). The patient does not want to believe in what is happening, there is a threat that it is necessary to deny to continue living. Thus, the informants speak about a future recovery.

This experiencing is congruent with findings obtained by Quill et al. [44], according to which one of the most important aspects to reach to the patient with advanced disease, would be the ability to change the trajectory of their illness.

The findings suggest that hope is a comfort factor, since patients trust that they still have some control over their health situation. Or even that it is possible to interfere with the course of the disease, as was also reported by other studies [45, 46].

According to Broggy [47], sometimes denial is so intense that resists victoriously to the reason evidence, under surprising hopes. Nevertheless, the body, through physical losses that are becoming more evident, restrictive and generators of impotence, indicates every day more clearly what will be the end.

As mentioned by Charmaz [48], there is a loss of identity, a loss of the “self”. Likewise, Cassel [49], described suffering as the state of discomfort induced by the person disintegration threat.

Physical losses and lack of autonomy favor the experiences of psycho-spiritual discomfort, since the self-esteem of the patient is affected.

Besides somatic vulnerability, informants are confronted with social vulnerability. The disease traps the patient and is a source of profound limitations, as the impossibility to return home.

The fact of sharing a room represents, for some patients, social comfort, letting them share their experience with other patients. For others, this is a discomfort factor since sharing a room deprives them of their freedom.

These findings are consistent with the study of Williams y Gardiner [50], which states that PCU should have collective and single rooms since the choice between these two types of room is not unanimous among patients.

These findings indicate that experienced losses and the feeling of powerlessness to solve them are the main source of discomfort experienced by the informants.

Indeed, according Kolcaba [10], one dimension of comfort is transcendence, defined as the state in which the person feels it has the potential to control their destiny, solve their problems.

The discomfort allows informants to intuit their finitude, which could lead them to the denial but also to a personal growth. There is no social or biological restriction that is so powerful that can overcome the freedom to take a stand, the freedom to choose what attitude to adopt in the face of suffering [51].

Thus, some informants choose to open up to spirituality, through intensification of the relationships and affections. Previous research in PC states that interpersonal relationships are strengthened at the end of life [52]. Nevertheless this study provides a new understanding by suggesting as comfort factors the intrapersonal relationships (making a recapitulation of his life and an evaluation of what is really important), interpersonal (with professional, family and friends) but also transpersonal relationships (with God and nature), since these relationships generate love towards themselves, others and the transcendent.

Indeed, Viktor Frankl [51], states that the core of the human being is the spirit, that is, the existence is always directed to something that is not only the very existence itself, but also a sense of life that must be met or someone to love.

This study supports the experience of comfort and discomfort as a balanced process, in which there is an oscillation between the losses and the valorisation of relationships. If in a sense discomfort prevails, in other the intensification of the affections predominates.

Even we mentioned the patient’s perspective, the majority of the interviewed (82 %) had cancer-related diagnoses, so the focus of the study was almost on palliative cancer patients. As pointed out in our introduction, ageing populations will lead to an increase in chronic conditions. Data indicates that these conditions could have a different dying trajectory than cancer [53].

So, grouping the experience of all PC patients together (cancer/non-cancer) does not take these differences in comfort experience into account.

We believe that our data covers the comfort experiences, but of course, the inclusion of more patients, with non-cancer diagnoses, and do the data analysis by separate in future research, might reveal additional relevant experiences.

## Conclusion

The experience of comfort, in the patient’s perspective, has been ignored by the literature on PC.

This study demonstrated that the PCU can be perceived as a space of comfort where the patient finds a suitable therapeutic context to their needs, but also as a place of discomfort where the patient is confronted with its vulnerability. It can be a space where there is a process of denial or openness to spirituality. The discomfort has underlying the experienced losses and the inability to transcend. The patient feels comfortable through the symptomatic control, compassionate care, the PCU differentiated environment, hope, interpersonal, transpersonal and intrapersonal relationships.

Even if such findings may seem intuitive, documenting them is crucial because it invites the reader to reflect on their beliefs about what it means to be comfortable for these patients, and allows the incorporation of this information in the design of focused interventions to maximize the comfort experience. Unless one offers patients the opportunity to be heard on their experience, their perspective will remain hidden and you could hardly provide comfort to them.

In addition, the findings provide useful information that leads us to two major future research lines: the need to develop and implement comfort interventions adapted and adjusted to these patients’ comfort needs; and the need to validate cross-culturally, to the contexts in study, an instrument for evaluating comfort, in order to assess the comfort interventions implemented.

## Abbreviations

PCU, palliative care unit; PC, palliative care; COREQ, cOnsolidated criteria for reporting qualitative research.

## Additional file

Additional file 1: COnsolidated criteria for REporting Qualitative research (COREQ): a 32-item checklist for interviews and focus groups. (DOCX 17 kb)
