# Supplementary material for: Comfort experience in palliative care: a phenomenological study
Source: BMC Palliat Care. 2016 Aug 2;15:71. doi: 10.1186/s12904-016-0145-0 (PMC4971655; doi:10.1186/s12904-016-0145-0)
Supplement: Additional file 1: — COnsolidated criteria for REporting Qualitative research (COREQ): a 32-item checklist for interviews and focus groups. (DOCX 17 kb) [file 12904_2016_145_MOESM1_ESM.docx]

**COnsolidated criteria for REporting Qualitative research (COREQ): a 32-item checklist for interviews and focus groups**

**Title:** COMFORT EXPERIENCE IN PALLIATIVE CARE: A PHENOMENOLOGICAL STUDY

**Authors:** AC, VP, MEB, JA

| **No** | **Item** | **Guide questions/description Response** | **Response** |
| --- | --- | --- | --- |
| **DOMAIN 1: Research team and reflexivity** | | | |
| **Personal Characteristics** | | | |
| 1 | Interviewer/facilitator | Which author/s conducted the interview or focus group? | AC |
| 2 | Credentials | What were the researcher's credentials? E.g. PhD, MD | AC - RN, MD  VP - RN, MD  MEB - RN, PhD  JA - RN, PhD |
| 3 | Occupation | What was their occupation at the time of the study? | AC - RN, PhD Student  VP -RN, PhD Student  MEB - RN, Coordinating Professor  JA- RN, Coordinating Professor |
| 4 | Gender | Was the researcher male or female? | AC - Female  VP, JA, MEB - Male |
| 5 | Experience and training | What experience or training did the researcher have? | AC – Health research MD  VP – Health research MD  MEB - Coordinating Professor in health research MD  JA – Qualitative researcher |
| **Relationship with participants** | | | |
| 6. | Relationship established | Was a relationship established prior to study commencement? | No. The participants were not acquainted to the researchers prior to the study commencements. |
| 7. | Participant knowledge of the interviewer | What did the participants know about the researcher? e.g. personal goals, reasons for doing the research | The participants knew that the aim of the study was to understand their experience of comfort and discomfort in the palliative care unit.  The participants were made aware that the interviewer was a nurse in the Spanish Center (in other unit) and the participants were also made aware that the interviewer was conducting this study as part of their PhD project. |
| 8. | Interviewer characteristics | What characteristics were reported about the interviewer/facilitator? e.g. Bias, assumptions, reasons and interests in the research topic |  |
| **DOMAIN 2: study design Theoretical framework** | | | |
| **Theoretical framework** | | | |
| 9. | Methodological orientation and Theory | What methodological orientation was stated to underpin the study? e.g. grounded theory, discourse analysis, ethnography, phenomenology, content analysis | Phenomenology descriptive. |
| **Participant selection** | | | |
| 10. | Sampling | How were participants selected? e.g. purposive, convenience, consecutive, snowball | Participants were recruited through purposive sampling. |
| 11. | Method of approach | How were participants approached? e.g. face-to-face, telephone, mail, email | Participants were approached face-to-face first by heads nurses of palliative care units, and after by the research interviewer |
| 12. | Sample size | How many participants were in the study? | 17 in total (10 participants Spanish palliative care unit, 7 participants Portuguese palliative care unit). |
| 13. | Non-participation | How many people refused to participate or dropped out? Reasons? | Non-participants refused to participate in the study. Non-participants dropped out during the interview. |
| **Setting** | | | |
| 14. | Setting of data collection | Where was the data collected? e.g. home, clinic, workplace | Interviews were conducted at a location that was convenient to the interviewee (their room or a intimate space in the palliative care units) |
| 15. | Presence of nonparticipants | Was anyone else present besides the participants and researchers? | No |
| 16. | Description of sample | What are the important characteristics of the sample? e.g. demographic data, date | Gender, age, time hospitalization and diagnostic are reported in Table 1 of the manuscript. |
| **Data collection** | | | |
| 17. | Interview guide | Were questions, prompts, guides provided by the authors? Was it pilot tested? | A pilot test with two patients was conducted in order to adjust the interview question. These interviews were not included. |
| 18. | Repeat interviews | Were repeat interviews carried out? If yes, how many? | No |
| 19. | Audio/visual recording | Did the research use audio or visual recording to collect the data? | Audio recording. |
| 20. | Field notes | Were field notes made during and/or after the interview or focus group? | No. |
| 21. | Duration | What was the duration of the interviews or focus group? | The mean interview duration was 32 minutes. |
| 22. | Data saturation | Was data saturation discussed? | Yes. Data collection ended when saturation was achieved. |
| 23. | Transcripts returned | Were transcripts returned to participants for comment and/or correction? | No, because of participants’ declining health.  Transcripts were reviewed by interviewer who listened to the audio recordings to verify their accuracy. |
| **DOMAIN 3: analysis and findings** | | | |
| **Data analysis** | | | |
| 24. | Number of data coders | How many data coders coded the data? | 2 researches (AC, VP) |
| 25. | Description of the coding tree | Did authors provide a description of the coding tree? | Yes (Figure I) |
| 26. | Derivation of themes | Were themes identified in advance or derived from the data? | Derived from the data. The sub-themes and themes were agreed upon by the analysis team through consensus. |
| 27. | Software | What software, if applicable, was used to manage the data? | QSR NVivo version 10 |
| 28. | Participant checking | Did participants provide feedback on the findings? | No, because of participants’ declining health. |
| **Reporting** | | | |
| 29. | Quotations presented | Were participant quotations presented to illustrate the themes / findings? Was each quotation identified? e.g. participant number | Yes |
| 30. | Data and findings consistent | Was there consistency between the data presented and the findings? | Yes |
| 31. | Clarity of major themes | Were major themes clearly presented in the findings? | Yes |
| 32. | Clarity of minor themes | Is there a description of diverse cases or discussion of minor themes? | Yes |
